# Good Health Practices and Well-Being among Adolescents with Type-1 Diabetes: A Cross-Sectional Study Examining the Role of Satisfaction and Frustration of Basic Psychological Needs

**DOI:** 10.3390/ijerph20031688

**Published:** 2023-01-17

**Authors:** Lika Hatzir, Rivka Tuval-Mashiach, Orit Pinhas-Hamiel, Tamar Silberg

**Affiliations:** 1 Department of Psychology, Bar-Ilan University, Ramat-Gan 52900, Israel; 2 Pediatric Endocrinology and Diabetes Unit, Edmond and Lily Safra Children’s Hospital, Sheba Medical Center, Tel-Hashomer, Ramat-Gan 52621, Israel; 3 Sackler School of Medicine, Tel-Aviv University, Ramat-Aviv 69978, Israel; 4 Department of Pediatric Rehabilitation, The Edmond and Lily Safra Children’s Hospital, Sheba Medical Center, Ramat-Gan 52621, Israel

**Keywords:** chronic illness, health behaviors, self-determination theory, psychological needs

## Abstract

Type 1 diabetes (T1D) is a chronic disease requiring medical adherence. However, among adolescents, non-adherence rates may reach up to 75%. Satisfaction or frustration with psychological needs is a crucial factor in the motivation and management of health-related behaviors. This study aimed to examine the differences in good health practices and psychological and physical well-being among adolescents with and without T1D and the mediating role of satisfaction and frustration of psychological needs on the association between good health practices and well-being in this population. A total of 94 adolescents (42 with T1D, 52 healthy controls, mean age 14.83 ± 1.82 years) completed questionnaires assessing good health practices, satisfaction or frustration of psychological needs, and well-being. Adolescents with T1D reported lower levels of physical well-being compared to healthy controls. Satisfaction or frustration of psychological needs had an effect on good health practices and psychological and physical well-being among healthy controls. Among adolescents with T1D, satisfaction or frustration of psychological needs was related to psychological well-being and partially related to physical well-being, but not to good health practices. The results demonstrate that the satisfaction or frustration of psychological needs has a unique effect on health behaviors and well-being among adolescents with T1D. This calls for further examination of the underlying mechanisms involved in health-related behaviors and well-being among adolescents with T1D.

## 1. Introduction

Type 1 diabetes (T1D), also known as “juvenile/childhood-onset diabetes,” is a serious, life-long autoimmune condition characterized by deficiency of insulin production [1]. Management of T1D requires a strict daily regimen of insulin injections, daily monitoring of blood sugar levels, and dietary restrictions. When unmanaged, T1D can lead to multisystem complications and an increased risk of serious, life-threatening health problems [1,2]. However, symptoms and complications of T1D can be reduced by intensive interventions including diet, physical activity, and drug therapy [1].

Type 1 diabetes can occur at any age but is most common between birth and 14 years [3]. Globally, there is a steady increase in incidence rate, with a peak of onset between 10 and 15 years [3]. Findings in Israel show that between 1997 and 2012 there was a 64% increase in T1D prevalence, with a peak occurrence between ages 10–14 [4], resulting in 12 new cases per year, per 100,000 children under 18 [5]. While medical adherence is a necessary component of effective illness self-management, it is an exceptionally difficult goal to achieve in the pediatric population, with reports of non-adherence rates among adolescents reaching up to 75% [6]. 

Adolescence is considered a transitional period between childhood and adulthood (i.e., the moratorium phase), in which identity evolves and responsibilities are gradually adopted. During this period, various processes take place to enable the formation of identity and a coherent psychological organization [7,8]. At the personal level, there is an increased focus on autonomy and individuation, formation of identity and a sense of capability, as well as emotional instability, risk-taking behaviors, and increased interest in the body [7]. At the interpersonal level, there is growing interest in the social world outside the family, separation from primary caregivers, tightening relationships with peers and feelings of relatedness, as well as the selection of romantic partners [7,9]. On the one hand, adolescence is a critical period for the development of good health patterns and practices (such as diet, physical activity, and substance use), while on the other hand, it is a period with elevated risk for adopting practices that can impact health and cause disease later in life [10].

Adolescents with T1D have to double the effort to form a coherent self-structure, since they must cope with both the challenges of puberty and the adjustment to life with a chronic illness [11,12]. Self-management behaviors are essential for children and adolescents with T1D (e.g., testing blood sugar, injecting insulin, following a diabetes meal plan, and engaging in physical activity) [13]. However, such behaviors often cause emotional distress, especially during peak-developmental stages [14]. Diabetes distress, for example, refers to the negative emotions that arise from living with diabetes [15]. Furthermore, feelings such as frustration, hopelessness, anger, guilt, and fear (all of which are common in individuals with T1D) are not considered psychiatric conditions; rather, they are viewed as an emotional response to the specific challenges of managing the illness [15]. Researchers have found that around one-third of adolescents with T1D experience severe diabetes distress, which strongly correlates with depressive symptoms. In addition, lower levels of self-efficacy and higher levels of negative thoughts and perceptions seem to be associated with greater diabetes distress [15]. Sadly, diabetes distress in adolescence has been associated with decreased adherence, poor motivation for diabetes management, and more health complications [14]. 

Self-determination theory (SDT) is an empirically derived theory of human motivation and psychosocial development that differentiates motivation in terms of being autonomous or controlled. SDT highlights intrinsic motivation and proposes that individuals tend to be driven by a need to grow and gain fulfillment [16,17]. The theory suggests that individuals are able to become self-determined when their needs for autonomy, competence, and relatedness are satisfied. For example, when the need for autonomy is satisfied, one often experiences a sense of volition, personal choice, and psychological freedom. However, when the need for autonomy is frustrated, one feels forced to act in certain ways by others. Similarly, competence satisfaction refers to feeling effective and capable of achieving desired goals, whereas competence frustration involves feelings of doubt and failure concerning efficacy. Lastly, relatedness satisfaction refers to the experience of intimacy and genuine connection with others, whereas relatedness frustration involves the experience of relational exclusion and loneliness [18].

When addressing chronic illnesses, SDT assumes that satisfaction of these basic needs will result in a more self-determined motivation for self-management. Motivation for self-management is considered to be scalar, running on a continuum from non-motivation (non-regulative behavior) via extrinsic motivation to intrinsic motivation. Studies focusing on T1D within an SDT framework found that adolescents with T1D who follow dietary recommendations under autonomous self-regulation maintain better self-care and metabolic control [19]. Furthermore, intrinsic motivation for T1D management has been shown to predict better diabetes management, glycemic control, and psychosocial functioning [20]. Despite the current knowledge, there is still lack of a complete understanding of the transitional processes to self-management in terms of how individuals integrate good health habits into their lives, particularly during adolescence, and how the sense of satisfaction or frustration of basic psychological needs may mediate the association between self-management of illness and well-being among adolescents with T1D [21]. 

**Figure 1:**
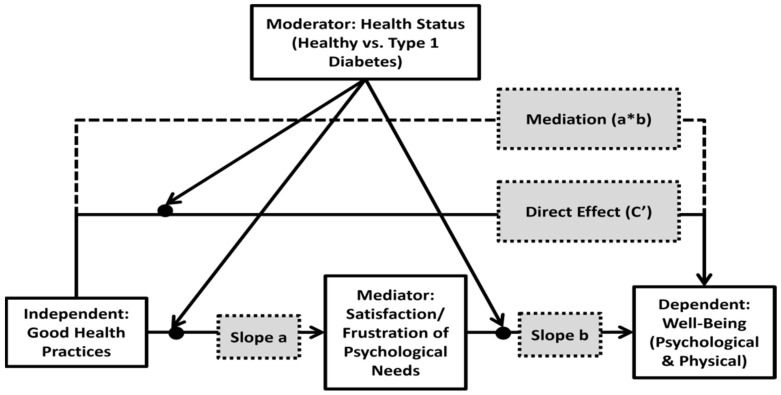
The study’s moderated–mediated model: the association between the independent variable (good health practices) and the dependent variable (adolescents’ perceived psychological and physical well-being) (direct effect (c’)) as mediated by satisfaction/frustration of psychological needs (Mediation (a*b)), and moderated by adolescent’s health condition.

Thus, the current study proposes a conceptual model examining psychological and physical well-being that is related to good health practices among adolescents with T1D (Figure 1). The main research aims were threefold: (1) to examine differences in good health practices and in psychological and physical well-being between adolescents with/without T1D; (2) to examine the association between good health practices and satisfaction and frustration of psychological needs (Figure 1: slope a); (3) and to examine the mediating effect of satisfaction and frustration of basic psychological needs on the association between good health practices and psychological and physical well-being among adolescents with/without T1D (Figure 1: moderation–mediation effects).

## 2. Methods

**Figure 2:**
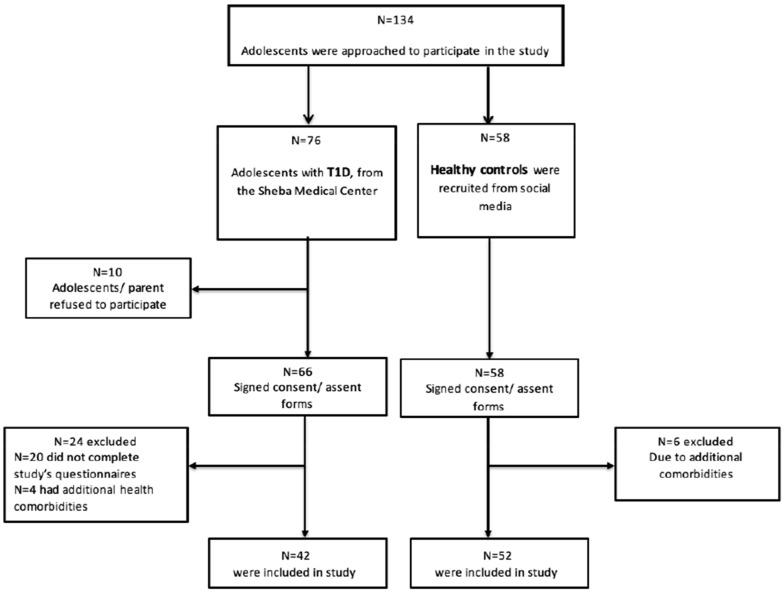
Study’s participants flow-diagram. Note: T1D: type-1 diabetes.

A total of 94 adolescents (ages 10–18 years) were recruited to participate in the study. Of these, 42 were diagnosed with T1D (mean ± SD age = 14.8 ± 2.06; 54.8% females) and 52 were healthy controls (mean age= 14.8 ± (1.63); 57.7% females). The T1D participants were recruited from the Pediatric Endocrinology and Diabetes Unit at the Edmond and Lily Safra Children’s Hospital, Sheba Medical Center, Israel, and the healthy controls were recruited from the general population via social media. A detailed advertisement (approved by the Ethical Review Board) was published on social media via Facebook and WhatsApp groups, describing the study’s purpose, requirements, need for parental consent and the researcher’s contact information for further details. In addition, participants notified that they will be reimbursed for their participation with a USD 10 gift card (see Figure 2 for participants’ flow-chart and Table 1 for a detailed description of sample characteristics).

Participants who agreed to participate were first screened for eligibility according to age and health condition. Parents of eligible participants were asked to give their consent for their child’s participation in the study. After receiving parental consent, questionnaires were sent to the adolescent via cellphone using a Qualtrics link. Adolescents assented to participate by agreeing to complete the study questionnaires. The study was approved by the Medical Center’s Ethical Review Board (6367-19-SMC). Date of approval: 7 July 2020.

### 2.1. Measures

#### 2.1.1. Independent Variables (Predictors)

Demographic information such as age, gender, education, parents’ marital status, as well as the relevant information regarding the medical condition (A1C levels, insulin pump treatments versus multiple daily injections, the use of a continuous glucose monitor, and illness duration) was collected via a short survey.

Good health practices: The Good Health Practices Scale [22] was used to examine general, non-illness related good health practices. The scale is a multidimensional health behavior checklist, meant to assess diverse health behaviors such as exercise, diet, smoking, information seeking, and other health behaviors (16 items). The items were rated on a 5-point Likert scale, ranging from 1 (not at all like me) to 5 (very much like me). Alpha reliability for the 16 items was 0.83. The scale was translated into Hebrew with the permission of the author.

#### 2.1.2. Mediating Variables

The Basic Psychological Need Satisfaction and Frustration Scale–general measure [23]. (24 items). The Satisfaction and Frustration Scale includes a balanced combination of satisfaction and frustration items regarding autonomy, competence, and relatedness. The items were rated on a 5-point Likert scale, ranging from 1 (completely disagree) to 5 (completely agree). The internal consistency estimates (Cronbach’s alpha) for the three subscales were shown to be above the acceptable range (α > 0.80). The scale was translated into Hebrew by Motty Benita [24].

#### 2.1.3. Dependent Variables (Outcomes)

KIDSCREEN-27 questionnaire [25]. The KIDSCREEN-27 questionnaire [25] was used in the current study to examine the outcome measure of psychological and physical well-being. The questionnaire consists of 27 items to assess 5 health-related quality of life dimensions. In the current study, only the two sub-scales evaluating physical and psychological well-being were included in the analysis as the study’s outcome measures. The items assess either the frequency of behavior/feelings or the intensity of an attitude and are answered on a five-point scale, from 1 (not at all) to 5 (always). The KIDSCREEN was translated into Hebrew with the author’s permission. Cronbach’s alpha coefficients were shown to be over 0.78 for all dimensions.

### 2.2. Statistical Analysis

Demographic and clinical characteristics are presented using descriptive statistics. Differences were assessed in the prevalence of background variables using chi-squared tests, and differences between groups in the dependent variables using independent *t*-tests. Associations between the predictors, mediators, and outcome variables were evaluated using Pearson correlations. Bonferroni correction was used to prevent inflated Type I error. Hierarchical linear regression models were applied, in which explanatory indicators were included in steps to assess the additional contribution of each variable [26]. First, the contribution of good health practices to satisfaction/frustration responses and the group difference in these two outcomes was tested. In this preliminary model, the second step included group interaction with health practices. In the final models, in which physical and psychological well-being were the outcomes, direct effects were tested in the first step, and the interaction effects were tested later. Lastly, a mediation model, was used to show the extent to which the indirect effect varied above and beyond the two groups. In cases of significant interactions, the interaction decompositions were presented graphically to show how the empirical slopes differ from each other. For these interaction analyses, the process procedure was used models 1, 59; [27].

## 3. Results

According to the first research aim, differences between groups in all study variables were examined. As can be seen in Table 2, adolescents in the healthy control group reported significantly higher levels of physical well-being (M = 3.65; SD = 0.76) compared to adolescents in the T1D group (M = 3.25; SD = 0.79) (t = 2.27, *p* < 0.05). Furthermore, adolescents in the T1D group reported higher levels of total satisfaction (M = 4.14; SD = 0.55) compared to adolescents in the healthy control group (M = 3.87; SD = 0.67) (t = −1.96, *p* < 0.05). In addition, scores on the competence satisfaction scale were higher among adolescents with T1D (M = 4.31; SD = 0.75) compared to healthy controls (M = 3.87; SD = 0.76) (t = −2.62, *p* < 0.05). 

An evaluation of specific variables possibly associated with illness-management behaviors among youth with T1D was conducted. Accordingly, the T1D group was divided into two sub-groups of above and below 7.5% A1C levels (i.e., average blood sugar levels over the past 3 months). A sub-analysis comparing participants in the two T1D groups was conducted on all outcome measures, with no significant differences observed. Furthermore, no significant correlations were found between illness duration and any of the outcome measures. Thus, all further analyses were conducted using the entire T1D group, in comparison to healthy controls.

To examine the second aim regarding the possible explanatory variables for the total-satisfaction and total-frustration indicators (slope “a” in the conceptual model [Figure 1]) a hierarchical regression analysis was conducted (Table 3). According to Table 3, the group main effect on total satisfaction was significant (β = 0.24, *p* < 0.05) and so was the main effect of good health practices (β = 0.40, *p* < 0.001), both contributing a total of 23% of the explained variance in total satisfaction. However, no main effect was found for the total frustration indicator. In step 2, the interactions between group and good health practices were found for both types of psychological needs (Satisfaction: β = −0.35, *p* < 0.05; Frustration: β = 0.35, *p* < 0.05) and added 7% to the total explanatory percent of both models. 

**Figure 3:**
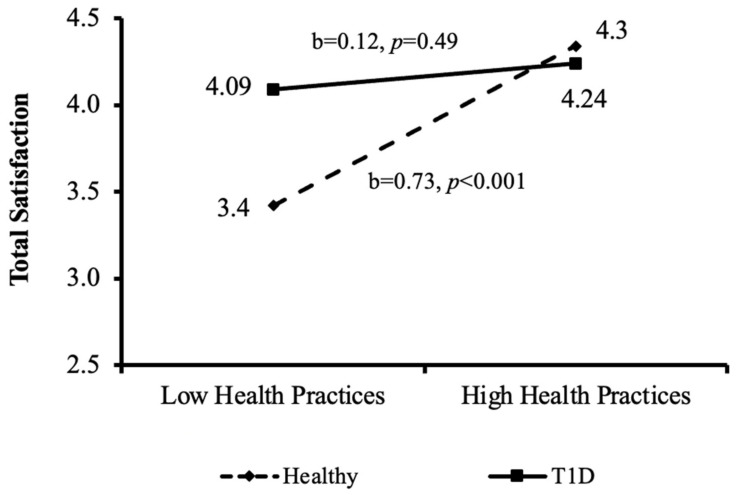
Interactions between the independent variable (good health practices) and the mediating variable (total satisfaction) by the moderating variable (health condition group); T1D= type–1 diabetes.

**Figure 4:**
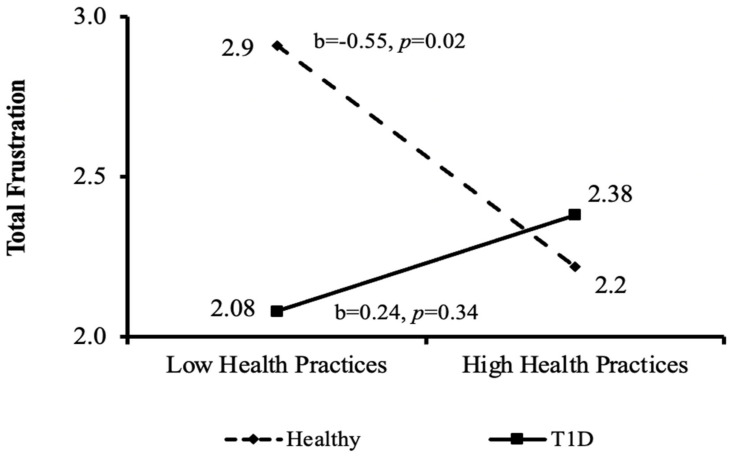
Interactions between the independent variable (good health practices) and the mediating variable (total frustration) by the moderating variable (health condition group). T1D = type-1 diabetes.

The interactions between good health practices and levels of total satisfaction and total frustration according to health condition groups (aim 2) are also depicted in Figure 3 and Figure 4. As can be seen in Figure 3, positive significant associations between good health practices and total satisfaction were found for the healthy control group (b = 0.73, *p* < 0.001), but not for the T1D group. 

As can be seen in Figure 4, significant negative associations between good health practices and total frustration were found for the healthy control group (b = −0.55, *p* < 0.02), but not for the T1D group. 

To examine the third aim regarding the mediating effect of satisfaction and frustration of basic psychological needs on the association between good health practices and well-being among adolescents with/without T1D. First, associations between the independent variables (good health practices), the mediators (satisfaction and frustration), and outcome variables (physical/psychological well-being) according to the two health condition groups (T1D vs. healthy controls) were conducted (Table 4). Second, a mediation model was applied (Table 5). Lastly, two hierarchical regression models (Table 6 and Table 7) were conducted to examine the study’s moderating–mediating model (Figure 1: moderation–mediation effects). 

The associations between the independent variables (good health practices), the mediators (satisfaction and frustration), and outcome variables (physical/psychological well-being) according to the two health condition groups (T1D vs. healthy controls) are presented in Table 4. As can be seen, good health practices were positively associated with both physical and psychological well-being only among healthy adolescents, but not among adolescents with T1D. Psychological well-being was positively associated with all satisfaction measures and negatively associated with all frustration measures, in both groups. However, among healthy controls, physical well-being was positively associated with all satisfaction measures, yet only negatively associated with relatedness frustration and total frustration measures. No other significant associations between physical well-being and frustration of psychological needs were found. In contrast, among adolescents with T1D, autonomy, competence and total satisfaction measures, but not relatedness satisfaction, were positively associated with physical well-being. In addition, only competence frustration and total frustration were negatively associated with physical well-being.

The indirect effects are presented in Table 5, which are beyond possible group moderation effects. A complete mediation effect was found in the path from satisfaction to physical well-being (indirect = 0.17, 95%CI [0.06, 0.32], and in the path from satisfaction to psychological well-being (indirect = 0.7, 95%CI [0.11, 0.48]. However, the mediation effect of frustration, above and beyond health condition groups was insignificant for both physical and psychological well-being.

Table 6 shows the effects of group, good health practices and total satisfaction and frustration measures on *physical well-being*. In Step 1 in both models, the group variable was a significant factor explaining physical well-being (β = −0.23, *p* < 0.05) and so was the health practices variable (β = 0.23, *p* < 0.05). However, both explained a relatively small percentage (8%) of the variance in physical well-being. In Step 2, the explanatory percent of total satisfaction (Model 1) or frustration (Model 2) was significant (ΔR2 = 0.13, *p* < 0.001; ΔR2 = 0.08, *p* < 0.05; respectively), explaining 23% and 18% of the variance in physical well-being, respectively. However, the interactions between group and health practices as well as group and satisfaction/frustration were insignificant (Step 3). 

**Figure 5:**
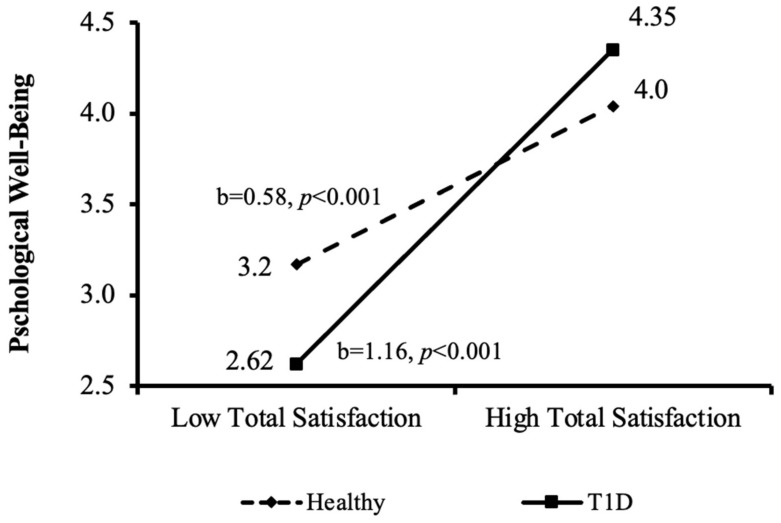
Interactions decomposition of the mediating variable (satisfaction) effect on the dependent variable (psychological well-being) according to the moderating variable (health condition groups). T1D = type 1 diabetes.

Table 7 shows the effects of group, good health practices and total satisfaction and frustration measures, with the outcome of *psychological well-being*. In Step 1, no group effect on psychological well-being was found. As expected, the good-health-practices variable was positively associated with psychological well-being (β = 0.25, *p* < 0.05). In Step 2, satisfaction (model 1) and frustration (model 2) showed strong significant associations with psychological well-being (β = 0.78, *p* < 0.001; β = −0.67, *p* < 0.001; respectively), and a large contribution to the explanatory percent of the models (ΔR2 = 0.47, *p* < 0.001; ΔR2 = 0.42, *p* < 0.001; respectively). In Step 3, the interaction between group and satisfaction showed an additional positive effect (β = 0.25, *p* < 0.05), which was further decomposed into two independent effects for the two groups (see Figure 5). As can be seen, the effect of total satisfaction on psychological well-being was twice as strong among the T1D group compared to the healthy control group, although it was positive in both (b = 1.16, *p* < 0.001; b = 0.058, *p* < 0.001; respectively).

## 4. Discussion

Adherence and self-management of a chronic illness are difficult at any age but are especially challenging during adolescence [28]. This study aimed to understand how self-management of good health practices can promote physical and psychological well-being among adolescents with T1D. Specifically, based on the SDT approach to motivation, the current study aimed to examine how satisfaction and frustration of psychological needs mediate the association between good health practices and well-being among adolescents with/without T1D.

In accordance with previous studies, it was found that adolescents with T1D reported lower levels of physical well-being (e.g., referring to general health, feeling fit and well, being physically active, full of energy, and being able to run) compared to healthy peers. This finding may be related to the fact that adolescents with T1D need to constantly monitor and balance their bodily responses (specifically their blood sugar levels) [29,30] to maintain good health and prevent deterioration. This may result in a lower sense of physical well-being, related to the burden of physically managing their medical condition. Furthermore, as there was no effect of A1C levels (i.e., average blood sugar levels over the past 3 months calculated as above or below 7.5%) on physical well-being, our finding may suggest that lower physical well-being is a broader consequence of T1D rather than a result of the balanced/unbalanced levels of blood sugar. In previous studies, the association between A1C levels and physical well-being was inconsistent, with some studies reporting a lack of association [31], and others supporting the association between these two variables [32,33], indicating a need to further explore the relationship between balanced blood-sugar levels and physical well-being among adolescents with T1D.

With regard to psychological well-being, adolescents with T1D did not report lower levels of psychological well-being (e.g., feeling life is enjoyable, being in a good mood, and having fun) compared to their peers. There is some inconsistency in the literature regarding levels of psychological well-being among adolescents with T1D. For example, a review paper focusing on suicide risk among adolescents with T1D found no differences in the percentage of fatal suicide acts between adolescents with T1D and healthy peers; however, adolescents with T1D reported a significantly higher rate (i.e., by more than two times) of suicidal thoughts compared to healthy peers [34]. In another meta-analytic review, adolescents with T1D were more likely to experience psychological difficulties such as depression and anxiety, compared to adolescents without T1D [35]. In contrast, the current study found that psychological well-being did not differ between the two groups. This finding can be understood according to previous studies indicating that the majority of adolescents with T1D report functioning well with regard to psychological well-being [36]. This is also in accordance with findings from a meta-analytic review showing that adolescents with T1D had more psychological difficulties, but this effect was small to medium and even smaller in studies published in recent years [35]. This latter finding is promising and may suggest that there is currently more awareness to the psychological challenges among children and adolescents with T1D, and that this awareness may be related to improved treatment approaches and to the increase in psychological well-being. 

Surprisingly, a positive association between good health practices and physical and psychological well-being was found only in the healthy control group. It is suggested that this finding may be explained by the mediating effect of satisfaction/frustration of psychological needs on the association between good health practices and well-being, which is elaborated below. 

It was found that compared to healthy peers, adolescents with T1D were overall more satisfied regarding the fulfillment of their psychological needs. Specifically, satisfaction with the need for competence was significantly higher among adolescents with T1D compared to healthy peers. This finding can be viewed in light of SDT, in which the task of managing good health in T1D (i.e., achieving glycemic control, managing daily insulin injections, measuring blood glucose, regulating carbohydrate intake, and keeping physical activity) is perceived as part of self-management behaviors that are necessary to staying healthy [33]. The higher levels of competence satisfaction in the T1D group are also in accordance with a previous review study [32] that addressed coping mechanisms of children and adolescents with chronic illness. In this study, the authors reported that adolescents with T1D who used effective control coping strategies had a better quality of life and lower A1C levels [32]. In addition, in a qualitative study conducted with adolescents with T1D, the perceived support of health care professionals and their acknowledgment and emphasis on adolescents’ success in keeping balanced levels of A1C were found to be perceived by adolescents as contributing to motivation and improved glycemic control [33]. Furthermore, these adolescents were also eager to share their strategies of diabetes self-management, emphasizing that the perceived competence and the acknowledgment of competence by the environment (i.e., health care professionals and parents) plays an important factor in self-management motivation [33]. This finding may emphasize the complexity and sensitivity needed from the environment in supporting well-being among adolescents with T1D. 

As the current study’s model suggests (Figure 1), total satisfaction of psychological needs was positively correlated with both physical and psychological well-being among healthy and T1D participants, while total frustration of psychological needs was negatively correlated with both physical and psychological well-being in both groups. These findings are in accordance with the SDT literature [16] and support the universality of the theory, so that a chronic medical condition (such as T1D) does not have a differential effect on the fundamental constructs of motivation. When considering chronic illnesses, special management issues arise, particularly around risk behaviors and medical regime [7]. In such cases, need support predicts more autonomous regulation of motivation, which in turn predicts better health outcomes [37]. The current findings also resonate with previous reports indicating that satisfaction of needs is linked to vitality and better physiological well-being, while frustration of needs is associated with physical symptoms and ill-being [38,39,40]. Furthermore, these findings are also in accordance with the biopsychosocial approach, which emphasizes that during adolescence, physical and psychological elements interact with external demands, making the achievement of tasks interdependent and in relation to each other [7]. 

When looking at each of the basic psychological needs independently, different patterns appeared. With regard to the need for autonomy, a positive correlation between autonomy satisfaction and psychological and physical well-being was found in both groups. Furthermore, frustration of the need for autonomy was negatively correlated with psychological well-being but not with physical well-being. This finding may be explained by the nature of autonomy–need frustration as defined by SDT, referring to individuals who feel in conflict or as being pressured to act [16,41], which are all crucial to psychological well-being, but not to physical well-being. This finding also supports the SDT distinction between *needs satisfaction* and *needs frustration,* suggesting that low levels of needs satisfaction might not be representative of high levels of needs frustration [42,43]. Thus, regardless of adolescents’ medical condition, the results support the SDT perspective underscoring the importance of autonomy–need satisfaction for psychological and physical well-being.

As for the need for competence, a positive correlation between competence-satisfaction and psychological and physical well-being was found in both groups. However, an interaction between the group and competence-need frustration was also found. Specifically, competence-need frustration was negatively correlated with psychological well-being in both groups; however, it was negatively associated with physical well-being only among adolescents with T1D, but not among healthy peers. This may be due to the relatively high maintenance load of health-related behaviors associated with chronic medical conditions, such as T1D. Furthermore, frustration of the need for competence may be related to the lack of support from health care professionals or parents, as perceived by adolescents with T1D. The literature indicates that parents of children and adolescents with T1D tend to act in a more controlling way toward their child’s illness-management behaviors, contributing to a lower sense of competency and lower physical well-being [33]. Furthermore, a meta-analysis focusing on the SDT perspective and its application to health contexts [44] found that the need for competence explained a larger proportion of the variances in health outcomes, compared to the need for autonomy. These findings, along with the current results, call for implementing a biopsychosocial approach for the treatment of adolescents with T1D, incorporating caregivers in support programs to help reduce levels of control and increase adolescents’ levels of trust and competency. 

Regarding the need for relatedness, a significant correlation was found in the healthy control group between satisfaction and frustration of the need for relatedness and between psychological and physical well-being. However, among adolescents with T1D, this correlation was only evident for psychological well-being. This may be explained by the fact that, according to SDT, relatedness is considered less central to intrinsic motivation for performing target behaviors [16]. Thus, as adolescents with T1D need to rely on their intrinsic motivation for achieving glycemic control and maintaining the health routines of diabetes, they may not associate relatedness as significant to their physical well-being. The current findings of the insignificant relationship between relatedness and physical well-being are in line with previous research [45] that found that the support of peers and parents was not predictive of better glycemic control and adherence to treatment. However, relatedness has been indicated as significantly important to psychological well-being, with findings showing that the stronger the emotional support from peers, the lower the level of diabetes distress [45]. This can be also supported by previous studies addressing the important role of recreational camps developed specifically for children and adolescents with diabetes, who often do not feel accepted and do not relate to their non-T1D peers [46]. Indeed, such camps have been reported to be supportive and to include a friendly and safe atmosphere, where the child/adolescent feels comfortable among other camp participants and staff who are coping with similar medical challenges [46]. 

When looking at the interactions between health practices, groups, and total satisfaction and frustration, it was found that among healthy adolescents, the higher the total-satisfaction, the higher the adolescent’s good health practice behaviors (Figure 2) and vice versa—the higher the total-frustration, the lower the good-health-practice behaviors (Figure 3). Surprisingly, this interaction was not found in the T1D group, in which good health practices were not related to the levels of total satisfaction or frustration. This finding can be explained by the results of a qualitative study that interviewed 13 adolescents regarding their self-management of diabetes [21]. One of the themes identified in the study was that keeping healthy was a major concern which was immediately impacted if blood sugar levels were not properly managed. As one of the participants explained: “from the years of doing it I know ……it will be my health that suffers ….". A second theme suggested that although keeping up with diabetes routines is an endless and exhausting process, there is a driving force to continue in order to keep healthy. To put it in the words of one of the participants: "Some days you just cannot be arsed to do anything … but you still do it" [21]. These themes may show that adolescents with T1D understand that maintaining their health-related routines is crucial for their survival (even if they do not feel like doing so), and thus continue to follow their health routines regardless of their level of satisfaction or frustration.

The major aim of the current study was to examine how satisfaction and frustration of psychological needs mediate the association between good health practices and well-being among adolescents with/without T1D. The theoretical model was partially supported with differences in the mediation-moderation effects between physical and psychological well-being. The results indicated that there were no significant interactions between group and health practices, and between group and total satisfaction/frustration on adolescents’ physical well-being (see Table 6). In contrast, a significant interaction was found between groups, satisfaction/frustration of psychological needs, and adolescents’ psychological well-being (see Table 7). Post hoc analysis of the source of the interaction was conducted, indicating that the association between total satisfaction and psychological well-being was twice as strong in the T1D group, compared to the healthy control group (see Figure 4). This finding is related to SDT and indicates the importance of complex psychological processes and factors involved in individuals’ motivation regarding their health and optimal functioning [44]. This may also reflect the constant challenge involved in balancing the medical condition of adolescents with T1D. It has been previously reported [47] that the perceived ability to manage diabetes was associated with higher levels of self- satisfaction. Furthermore, successful self-management reduced the felt sense of difficulty and interruption of adolescents’ resources [21]. This may represent the acceptance of diabetes as part of ones’ life [21], possibly resulting in higher satisfaction and higher psychological well-being. 

Finally, the mediation model of satisfaction/frustration of psychological needs was also partially supported. A complete mediation effect of satisfaction on the association between good health practices and physical/psychological well-being was found. However, the mediation effect of frustration, above and beyond health condition groups, was insignificant for both physical and psychological well-being (see Table 5). It was found that satisfaction of the three basic needs is most important for adjustment and well-being across different age groups, cultures, and situations [41,48]. As assumed, adolescents were found to be more satisfied with life in general and had a much more positive affect when their needs were satisfied [41,49,50]. A recent study [51] addressed the hypothesis that adolescents are proactive regarding their need satisfaction. The authors presented the term *need crafting*, referring to the tendency of people to create optimal conditions for their psychological growth and need satisfaction. Need crafting is composed of two main components: awareness of personal satisfaction sources (activities, relations, and contexts) and a tendency to act upon this awareness when making choices. The study found that adolescents who engaged in more need-crafting behaviors reported higher well-being, which was also associated with need satisfaction. This suggests a possible reciprocal relationship between need crafting, need satisfaction, and well-being: need crafting contributes to need satisfaction, which in turn is associated with psychological well-being. It was further suggested that good psychological well-being contributes to the availability of the vitality needed for further need crafting [51]. One of the possibilities that arises from this study is that need crafting can serve as a buffer against need frustration [52]. Nevertheless, it was also acknowledged that need crafting may vary between and within adolescents and may not represent a constant construct [51], possibly suggesting the inclusion of need-crafting strategies in treatment protocols for adolescents with T1D.

## 5. Limitations

The current study was subject to several limitations. First, the fact that the sample size was relatively moderate might have contributed to some of the null findings. Future studies should aim at examining the current theoretical model using larger samples. Second, in light of the biopsychosocial approach, the fact that only Israeli Jewish adolescents participated in this study, who were recruited from one pediatric endocrinology and diabetes department, might have contributed to a culturally related bias of satisfaction or frustration of psychological needs, or may have reflected the approach of the medical team in the department towards self-management of good health practices. Thus, it is recommended that future studies include various populations and collect information from more than one site. A third limitation was the decision to focus on adolescents’ subjective reports, that is, reports based on the adolescent’s point of view and perception. In order to understand the full picture, it may be helpful to rely also on parents, school staff, and health care practitioners. Fourth, the fact that the healthy participants were recruited via social media may create a credibility bias due to lack of adequate representation of the populations’ diversity, not reaching different participants subgroups [53,54,55]. Fifth, to examine the theoretical model, the current study used well established questionnaires that were used in previous studies. However, these questionnaires were not validated for the current sample (except for internal validity) due to the relatively small sample size. Future studies should aim at recruiting larger samples and conducting validity assessments to support the generalization of the findings to the Israeli population. Lastly, the current sample included only those adolescents who volunteered (agreed to devote time and resources) in order to participate in this study; thus, it may not represent the larger population of youth with T1D. This problem is also known as *self-selection bias*, in which study respondents who choose to participate do not always accurately represent the entire target population [56]. 

## 6. Conclusions

This study showed, for the first time, the importance of addressing the satisfaction and frustration of psychological needs when assessing the relationship between health practices and well-being among adolescents with chronic illnesses, such as T1D. It was found that adolescents with T1D report lower physical but not psychological well-being. Furthermore, the study was able to differentiate between factors associated with physical and psychological well-being, which are important specifically in a population dealing with a chronic illness. Another implication of this study is the important role of focusing on the positive aspects of adolescents’ health management and making an effort to support need satisfaction of autonomy, competence, and relatedness in order to improve well-being. Thus, it might be helpful to encourage awareness of the need to develop skills to help facilitate the development of self-regulation, and thereby increase need satisfaction and well-being. Additionally, it is important to notice that focusing on control and judgement of adolescents’ behaviors may not be useful and may contribute to the development of external, but not internal, self-regulation [37]. Lastly, the current study calls for a further emphasis on strengthening the understanding of clinicians and caregivers regarding the importance of the SDT perspective for supporting the association between good health practices and well-being in the T1D population.

## Figures and Tables

**Table 1:** Descriptive statistics of sample characteristics.

	Healthy Controls	N	Type 1 Diabetes	N	df	X^2^	*p*-Value
							
Gender (%; Female)	57.7	30	54.8	23	1	0.08	0.78
Age; Mean (SD)	14.85 (1.63)	52	14.81 (2.06)	42	92	t = 0.10	
							
Marital status of the parents							
Married (%)	86.5	45	83.3	35	1	1.45 ^1^	0.23
Divorced (%)	13.5	7	14.3	6			0.38
Single parent (%)	0	0	2.4	1			
							
Using Insulin Pump	N/A	N/A	69.0	29			
Using Continuous Glucose Monitoring device	N/A	N/A	71.4	30			
Illness duration in years (range)	N/A	N/A	4.5 (1–15)	40			

Note: N/A = not applicable; SD = standard deviation; df = degree of freedom. ^1^ Married versus all other marital statuses, second *p*-value for Fisher Exact test.

**Table 2:** Descriptive statistics of research variables followed by group comparisons.

	Healthy Means (SD)	Type-1 Diabetes Means (SD)	Actual Range	t	*p*-Value	α (Cronbach’s Alpha)
**Physical well-being**	3.65 (0.76)	3.25 (0.79)	1.60–5.00	2.27 *	0.03	0.784
**Psychological well-being**	3.59 (0.63)	3.77 (0.82)	1.43–5.00	−1.10	0.28	0.885
**Good health practices**	3.12 (0.56)	3.20 (0.55)	1.40–5.20	−0.62	0.54	0.714
**Total satisfaction**	3.87 (0.67)	4.14 (0.55)	2.50–5.00	−1.96 ^§^	0.05	0.861
**Total frustration**	2.55 (0.66)	2.26 (0.97)	1.08–5.00	1.63	0.11	0.860
**Autonomy satisfaction**	3.69 (0.80)	3.96 (0.67)	2.25–5.00	−1.63	0.11	0.714
**Relatedness satisfaction**	4.06 (0.77)	4.16 (0.76)	2.00–5.00	−0.58	0.56	0.778
**Competence satisfaction**	3.87 (0.76)	4.31 (0.75)	2.00–5.00	−2.62 *	0.01	0.821
**Autonomy frustration**	2.88 (0.87)	2.56 (1.05)	1.00–5.00	1.52	0.13	0.754
**Relatedness frustration**	2.35 (0.85)	2.13 (1.09)	1.00–5.00	1.08	0.29	0.750
**Competence frustration**	2.65 (0.85)	2.33 (1.23)	1.00–5.00	1.39	0.17	0.821

Note: ^§^ *p* = 0.05; * *p* < 0.05.; SD= standard deviation.

**Table 3:** Hierarchical regression for the mediating variables (Total Satisfaction and Total Frustration outcomes).

	TOTAL-SATISFACTION		TOTAL-FRUSTRATION	
	Coefficient	*p*-Value	Coefficient	*p*-Value
**Step 1: Main Effects**				
Group: Healthy (0) vs T1D (1)	0.24 *	0.03	−0.21	0.08
Good health practices	0.40 ***	<0.001	−0.13	0.27
F	10.28 ***	<0.001	2.41	0.10
df	2.71		2.71	
R^2^	0.23 ***	<0.001	0.06	0.10
**Step 2: Interaction Effects**				
Interaction Group x Good health practice	−0.35 *	0.01	0.35 *	0.02
Healthy	b = 0.73 ***	<0.001	b = −0.55 *	0.02
T1D	b = 0.12	0.49	b = 0.24	0.34
ΔR^2^	0.07 *	0.01	0.07 *	0.02
F	9.63 ***	<0.001	3.49 *	0.02
df	3.70		3.70	
R^2^	0.29 ***	<0.001	0.13 *	0.02

Note: * *p* < 0.05; *** *p* < 0.001; T1D: type-1 diabetes; DF = degree of freedom.

**Table 4:** Correlations between the independent (good health practices), the mediating (Satisfaction and frustration of basic psychological needs) and the dependent (physical and psychological well-being) variables according to the moderating variable (research groups).

	HEALTHY (N = 42)		TYPE 1-DIABETES (N = 38)	
	Physical Well-Being (*p*-Value)	Psychological Well-Being (*p*-Value)	Physical Well-Being (*p*-Value)	Psychological Well-Being (*p*-Value)
**Good health practices**	0.46 ** (0.003)	0.52 *** (<0.001)	−0.02 (0.93)	0.002 (0.99)
				
**Total-Satisfaction**	0.48 ** (0.001)	0.70 *** (<0.001)	0.39 * (0.02)	0.77 *** (<0.001)
**Total-Frustration**	−0.32 * (0.04)	−0.75 *** (<0.001)	−0.33 (0.05)	−0.67 *** (<0.001)
**Autonomy satisfaction**	0.33 * (0.03)	0.67 *** (<0.001)	0.37 * (0.02)	0.50 ** (0.002)
**Relatedness satisfaction**	0.46 ** (0.002)	0.60 *** (<0.001)	0.19 (0.26)	0.54 *** (<0.001)
**Competence satisfaction.**	0.45 ** (0.003)	0.52 *** (<0.001)	0.35 * (0.03)	0.73 *** (<0.001)
**Autonomy frustration**	−0.17 (0.29)	−0.70 *** (<0.001)	−0.20 (0.23)	−0.48 ** (0.002)
**Relatedness frustration**	−0.39 * (0.01)	0.64 *** (<0.001)	−0.24 (0.15)	−0.63 *** (<0.001)
**Competence frustration**	−0.17 (0.29)	−0.40 *** (<0.001)	−0.41 * (0.01)	−0.61 *** (<0.001)

Note: * *p* < 0.05; ** *p* < 0.01; *** *p* < 0.001.

**Table 5:** Indirect effects of Satisfaction/Frustration of psychological needs (mediator) on the relationship between good health practices (independent) and physical/psychological well-being (dependent).

Independent (I)	Mediator (M)	Dependent (D)	I → M	M → D	I → D	Indirect	95%CI Indirect
Good health practices	Satisfaction	physical well-being	0.33 ** (0.11)	0.52 *** (0.12)	0.11 (0.14)	0.17 * (0.06)	[0.06, 0.32]
Good health practices	Frustration	physical well-being	−0.18 (0.14)	−0.28 ** (0.09)	0.23 (0.14)	0.05 (0.06)	[−0.03, 0.19]
Good health practices	Satisfaction	psychological well-being	0.33 ** (0.11)	0.82 *** (0.08)	−0.02 (0.10)	0.27 * (0.09)	[0.11, 0.48]
Good health practices	Frustration	psychological well-being	−0.18 (0.14)	−0.62 *** (0.06)	0.14 (0.09)	0.11 (0.11)	[−0.08, 0.37]

*** *p* < 0.001, ** *p* < 0.01, * *p* < 0.05. Standard errors in parentheses, CI = confidence interval in squared brackets.

**Table 6:** Hierarchical regression models for the dependent variable *physical well-being*.

	Model 1		Model 2	
	Coefficient	*p*-Value	Coefficient	*p*-Value
**Step 1: Main Effects**				
Group: Healthy (0) vs T1D (1)	−0.23 *	0.04	−0.23 *	0.04
Good health practices	0.23 *	0.04	0.23 *	0.04
F	3.94 *	0.02	3.94 *	0.02
df	2.71		2.71	
R^2^	0.08 *	0.02	−0.23 *	0.04
**Step 2: Mediation Effects**				
Satisfaction/Frustration	0.41 ***	<0.001	−0.29 *	0.01
ΔR^2^	0.13 ***	<0.001	0.08	0.08
F	7.00 ***	<0.001	5.07 **	0.003
df	3.70		3.70	
R^2^	0.23 ***	<0.001	0.18 **	0.003
**Step 3: Interaction Effects**				
Group X Good health practices	−0.20	0.23	−0.23	0.15
Group X Satisfaction/Frustration	0.06	0.71	−0.06	0.79
ΔR^2^	0.02	0.48	0.03	0.35
F	4.46 **	0.001	3.47 **	0.008
df	5.68		5.68	
R^2^	0.25 **	0.001	0.20 **	0.008

Note: Model 1 refers to total satisfaction as the mediating variable and Model 2 refers to total frustration as the mediating variable. * *p* < 0.05. ** *p* < 0.01. *** *p* < 0.001; T1D = type 1 diabetes; DF= degree of freedom.

**Table 7:** Hierarchical regression models for the dependent variable psychological well-being.

	Model 1		Model 2	
	Coefficient	*p*-Value	Coefficient	*p*-Value
**Step 1: Main Effects**				
Group: Healthy (0) vs T1D (1)	0.12	0.29	0.12	0.29
Good health practices	0.25 *	0.03	0.25 *	0.03
F	3.11 ^§^	0.05	3.11 ^§^	0.05
df	2.71		2.71	
R^2^	0.06 ^§^	0.05	0.06 ^§^	0.05
**Step 2: Mediation Effects**				
Satisfaction/Frustration	0.78 ***	<0.001	−0.67 ***	<0.001
ΔR^2^	0.47 ***	<0.001	0.42 ***	<0.001
F	28.43 ***	<0.001	22.96 ***	<0.001
df	3.70		3.70	
R^2^	0.55 ***	<0.001	0.50 ***	<0.0001
**Step 3: Interaction Effects**				
Group X Good health practices	−0.15	0.20	−0.06	0.63
Group X Satisfaction/Frustration	0.31 **	0.007	0.05	0.74
Healthy	b = 0.58 ***	<0.001	−	−
T1D	b = 1.16 ***	<0.001	−	−
ΔR^2^	0.05 *	0.03	0.003	0.80
F	20.01 ***	<0.001	13.56 ***	<0.001
df	5.68		5.68	
R^2^	0.60 ***	<0.001	0.50 ***	<0.001

Note: Model 1 refers to total satisfaction as the mediating variable and Model 2 refers to total frustration as the mediating variable. T1D: type-1 diabetes; DF= degree of freedom; ^§^ *p* = 0.05. * *p* < 0.05. ** *p* < 0.01. *** *p* < 0.001.

## Data Availability

The data that support the findings of this study are available from the corresponding author, (T.S.), upon reasonable request.
